# Mycophenolate Mofetil Versus Prednisone for Induction Therapy in Steroid-Sensitive Idiopathic Nephrotic Syndrome in Children: An Observational Study

**DOI:** 10.1016/j.xkme.2023.100776

**Published:** 2023-12-10

**Authors:** Alexandra Mazo, Stella Kilduff, Tanya Pereira, Sonia Solomon, Robin Matloff, Anna Zolotnitskaya, Dmitry Samsonov

**Affiliations:** 1 Pediatric Nephrology Division, Maria Fareri Children’s Hospital, Westchester Medical Center, Boston Children’s Health Physicians, New York Medical College, Valhalla, New York; 2 Pediatric Nephrology Division, Ann and Roberst H. Lurie Children’s Hospital of Chicago, Northwestern University Feinberg School of Medicine, Chicago, Illinois; 3 Pediatric Nephrology Division, Connecticut Children’s, University of Connecticut School of Medicine, Hartford, Connecticut; 4 Pediatric Nephrology Division, The Children’s Hospital at Montefiore, Albert Einstein College of Medicine, Bronx, New York

**Keywords:** Minimal change disease, prednisone, relapse, mycophenolate mofetil, induction

## Abstract

**Rationale & Objective:**

High-dose steroids are recommended for the induction of idiopathic nephrotic syndrome. The aim of this study was to compare standard induction therapy with Mycophenolate Mofetil (MMF). We hypothesized that MMF could be noninferior to steroids in maintaining steroid-induced remission. The second aim was to reduce steroid-induced side effects.

**Study Design:**

This was an observational study.

**Setting & Population:**

Patients 2-11 years with first episode of nephrotic syndrome who entered remission within 2 weeks of standard steroid treatment were eligible for enrollment. Patients in the experimental group completed 12-week induction with MMF, whereas the control group continued a standard 12-week steroid protocol.

**Exposures:**

MMF and prednisolone were used in the study.

**Outcomes:**

The primary study outcomes were relapse rate and relapse-free interval during a 52-week follow-up.

**Analytical Approach:**

Descriptive statistics were used for analysis.

**Results:**

Ten of 41 eligible patients consented to participate in the MMF group and 8 completed the study. The control group included 31 patients, with 23 patients who completed 52 weeks follow-up. During the induction phase, 3 out of 10 patients (30%) in the MMF group and 1 out of 31 (3%) in the control group (*P* = 0.04) developed relapse. During the 52 weeks follow-up period, 7 out of 10 patients (70%) in the MMF group and 19 out of 31 (61%) in the control group developed relapse (*P* = 0.72). The median relapse-free interval was 11 and 19 weeks in MMF and control groups, respectively (*P* = 0.60). No serious side effects were recorded in either group.

**Limitations:**

The limitations of the study were low patient numbers receiving MMF and single-center design.

**Conclusions:**

Our small cohort of patients treated with MMF reported a higher relapse rate during the induction phase. However, by 12 months of follow-up the relapse rate and relapse-free intervals were similar between both groups. All patients tolerated MMF without significant side effects, and those who relapsed remained steroid-sensitive.

## Plain-Language Summary

Despite their known side effects steroids remain a standard induction therapy for new onset nephrotic syndrome in children. The aim of this study was to assess whether mycophenolate mofetil (MMF) can be as successful as steroids in maintaining steroid-induced remission. Patients who achieved remission within 2 weeks of steroid treatment had either continued steroids for an additional 10 weeks or switched to MMF. The results of the study showed a higher relapse and infection rate in the MMF group. By 12 months there were no differences in the relapse rate and relapse-free interval between the groups. Larger multicenter studies are underway to demonstrate noninferiority of MMF to steroids in steroid-sensitive nephrotic syndrome.

## Graphical abstract

**Figure:**
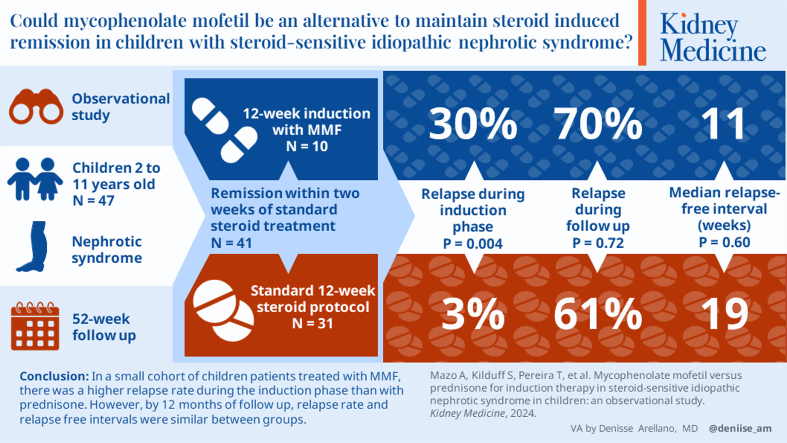

Steroid-sensitive nephrotic syndrome is the most common form of nephrotic syndrome in children. About 80%-90% of children with primary nephrotic syndrome aged 2-12 years respond to treatment with steroids, usually within the first 2 weeks of treatment.^1^ Kidney biopsy performed at presentation is consistent with minimal change histopathology. Steroid responsiveness remains the main prognostic factor for the preservation of kidney function, whereas the biopsy result does not change the treatment of this category of patients. Therefore, kidney biopsy is reserved mainly for those children whose nephrotic syndrome is resistant to steroids. The usual length of treatment is 8-12 weeks of high-dose steroids resulting in cumulative exposure of prednisone ∼120 mg/kg.^2^^,^^3^ This exposure carries a significant risk of unwanted treatment-related side effects, such as increased appetite, obesity, striae, behavior and personality changes, increased risk of infections, and others.^2^^,^^4^, ^5^, ^6^, ^7^, ^8^, ^9^ Sometimes 60%-80% of children may experience relapses after the initial course of steroids resulting in further steroid exposure and accumulation of unwanted effects, including growth retardation, hypertension, bone demineralization, and cataracts.^10^^,^^11^

The induction treatment of steroid-sensitive nephrotic syndrome with this dose and duration of steroids was established in the 1960s to 1970s.^1^^,^^12^ Over the last decades, shorter induction courses of steroids have been used in steroid-sensitive patients with acceptable outcomes.^13^^,^^14^ By contrast, longer steroid induction has not resulted in better long-term outcomes.^15^^,^^16^ One study that evaluated induction therapy of steroids combined with cyclosporine versus standard steroid protocol reported no benefit in relapse rate after initial 9-12 months.^17^ To the best of our knowledge, no additional randomized trials using other medications in the induction phase have been reported to date.^3^^,^^10^^,^^11^ There is an ongoing trial that compares induction with steroids to a combination of steroids and levamisole^18^ and another study comparing steroid induction to mycophenolate mofetil (MMF).^19^ Zhang et al^20^ published a report of 3 nephrotic syndrome pediatric cases successfully treated with Rituximab alone as an induction therapy.

The current observational study proposes to replace steroid therapy with MMF for completion of the induction phase of treatment in children with steroid-sensitive nephrotic syndrome. The rationale is that MMF has had considerable success in preventing relapses of frequently relapsing nephrotic syndrome and steroid-dependent nephrotic syndrome.^21^^,^^22^ We hypothesized that MMF can be noninferior to steroids in maintaining steroid-induced remission. The second aim was to mitigate side effects typically associated with prolonged steroid usage.

## Methods

### Study Design

**Figure 1:**
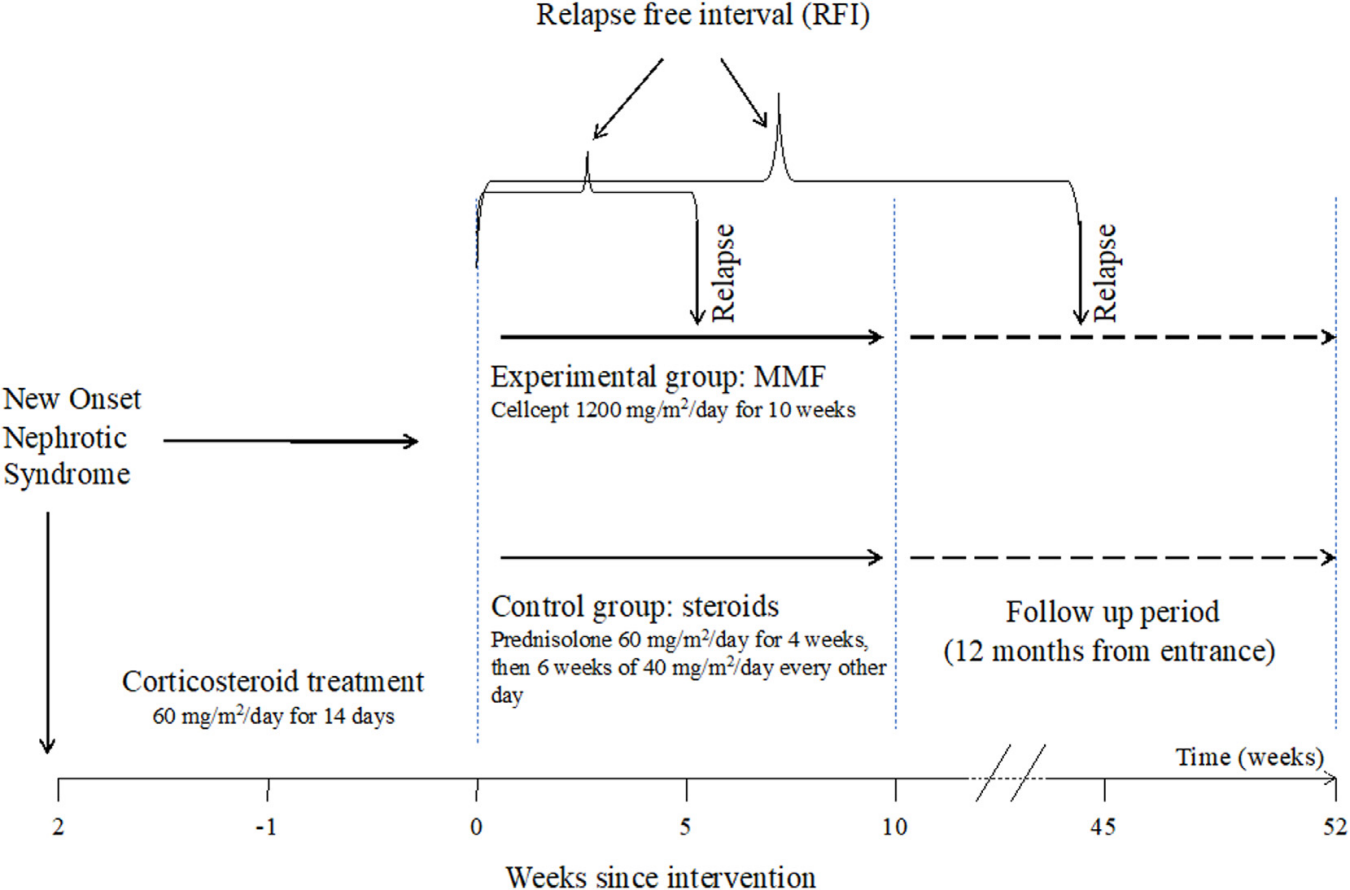
Study design. MMF, mycophenolate mofetil.

This was a prospective single-center observational study (Fig 1). Patients were enrolled in pediatric nephrology clinic of Boston Children’s Health Physicians, New York Medical College (NYMC). This study was approved by institutional review board (Protocol #10946). Consent for study participation was obtained for all patients.

### Patient Selection

Children aged 2-12 years who presented for the first time with a clinical and laboratory diagnosis of nephrotic syndrome (see definitions) and responded to standard corticosteroid (prednisone or prednisolone) therapy 60 mg/m^2^/d within 2 weeks were eligible to participate in the study. Exclusion criteria were gross hematuria, decreased complement C3 level, or participation in another study.

### Study Protocol

All patients were offered to complete their induction course with 10 weeks of MMF therapy (brand CellCept) suspension 600 mg/m^2^/dose twice daily (experimental group). Patients who did not consent to be switched to MMF treatment entered a control group of the study by completing 10 additional weeks of standard corticosteroid induction (additional 4 weeks of prednisolone 60 mg/m^2^/d daily and then 6 weeks of 40 mg/m^2^/d every other day).

Relapse (see definitions) during induction therapy with MMF was considered a treatment failure, and MMF therapy was discontinued immediately. All patients experiencing relapse (either during or after completion of MMF therapy) were treated with standard therapy with prednisone or prednisolone 60 mg/m^2^/d until remission was achieved. After achieving remission, the treating pediatric nephrologist was free to employ whatever treatment he or she felt was clinically indicated.

The first primary outcome measure of this study was the time to relapse during the induction phase of treatment. Relapse-free intervals and relapse rates were calculated. Secondary outcome measures included side effects of MMF and steroids: weight gain, hypertension, leukopenia, infection rate, gastrointestinal (GI) side effects, and hospitalizations.

### Definitions^3^^,^^23^

1. Nephrotic syndrome: triad of edema, hypoalbuminemia (serum albumin level below 2.5 g/dL) and nephrotic-range proteinuria (urine protein-to-creatinine ratio above 2 mg/mg)
2. Steroid-sensitive nephrotic syndrome: a confirmed remission within 14 days of initial treatment with oral steroids at a dose of 60 mg/m^2^/d
3. Frequently relapsing nephrotic syndrome: 4 or more relapses within 1 year or 2 or more relapses within 6 months
4. Steroid-dependent nephrotic syndrome: relapse during treatment with steroids or within 2 weeks after discontinuation of steroids
5. Remission: urine negative or trace by Albustix (parent home testing) for 3 consecutive days confirmed by a urine protein-to-creatinine ratio of equal or less than 0.3 mg/mg
6. Relapse: urine protein 2+ or more by Albustix for 3 consecutive days confirmed by urine protein-to-creatinine ratio above 2 mg/mg
7. Treatment failure: relapse during induction treatment with MMF or steroids
8. Relapse-free interval: time from the study entrance to the first relapse
9. Hypertension: Blood pressure > 95% for sex and height on 3 measurements obtained on different days

Data collected from May 2013 to October 2022 included age, sex, weight, height, blood pressure, urinalysis, urine protein-to-creatinine ratio, CBC, infections, antihypertensive medications, antibiotics, hospitalizations, and GI problems.

### Statistical Analysis

Shapiro-Wilk test was used to assess normality of data distribution. Categorical variables are presented as numbers (%). Continuous variables are presented as mean ± SD or median (interquartile range [IQR]). Univariate associations with outcomes were compared by Fisher exact test for categorical variables and *t* test or Mann-Whitney *U* test for continues variables. We performed intention-to-treat and per-protocol analyses.

## Results

Forty-seven children 2-12 years of age with a new diagnosis of nephrotic syndrome were seen in our practice between 2013 and 2021. Forty-one out of 47 achieved remissions within 2 weeks and were eligible for participation (Table 1). Ten of them consented to participate in the MMF group and 8 completed the study. The control group included 31 patients, with 23 patients who completed 52 weeks follow-up (Fig S1). Nine patients transferred their care out of our network with a median follow-up of 2 months, IQR (2-6). A patient in the MMF group was started on MMF while he had proteinuria with UPC 0.4 mg/mg and reported a relapse during induction. All patients were generally healthy and did not have any major comorbid conditions other than mild asthma.

**Table 1:** Patients’ Characteristics During Induction (ITT Population)

	MMF Group (n = 10)	Control Group (n = 31)	*P*
**Age (y), Me (IQR)**	4.2 (3-9)	4.4 (3-6)	0.90
**Male sex, n (%)**	9 (90)	18 (58)	0.12
**Hypertension, n (%)**	0	3 (9.6)	0.56
**Infections, n (%)**	5 (50)	4 (13)	0.01^a^
**Antibiotics, n (%)**	2 (20)	0	0.05
**Δ BMI, Me (IQR)**	0.1 (−0.4 to 0.9)	0.2 (−0.6 to 0.9)	0.96
**Δ Weight (kg), Me (IQR)**	1.2 (0.4-1.8)	1 (0-2.1)	0.66

Abbreviations: BMI, body mass index; ITT, intention-to-treat; IQR, interquartile range; Me, median; MMF, mycophenolate mofetil.

a *P* < 0.05.

**Figure 2:**
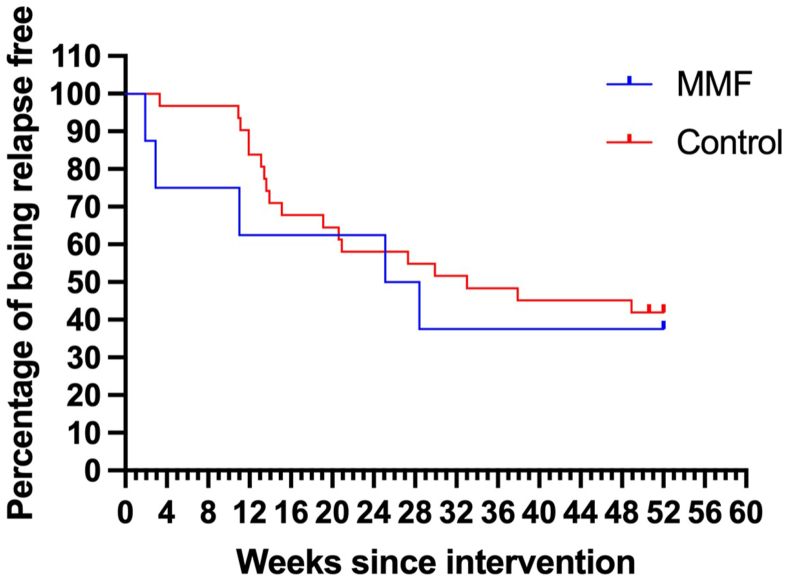
Relapse-free interval by Kaplan-Meier analysis, ITT population (logrank test *P* = 0.60). ITT, intention-to-treat; MMF, mycophenolate mofetil.

During the induction phase, 3 out of 10 patients (30%) in the MMF group and 1 out of 31 (3%) in control group (*P* = 0.04) developed relapse (Table 2). Two patients developed relapse during upper respiratory infections, the third was asymptomatic. During the follow-up period (52 weeks), 7 out of 10 patients (70%) in the MMF group and 19 out of 31 (61%) in the control group developed relapse (*P* = 0.72). The median relapse-free interval was 11 (IQR, 4-18) and 19 (IQR, 13-33) weeks in MMF and control groups, respectively (*P* = 0.60) (Fig 2). During 52 weeks of study the median relapse rate was 2.5 (IQR, 0-3) in the MMF group and 1 (IQR, 0-3) in the control group (*P* = 0.42) (Table 2).

**Table 2:** Nephrotic Syndrome Course in ITT Population

	MMF Group (n = 10)	Control Group (n = 31)	*P*
**Patients with relapses during induction, n (%)**	3 (30)	1 (3)	0.04^a^
**Patients with relapses during 52-wk follow-up, n (%)**	7 (70)	19 (61)	0.72
**Relapse rate, Me (IQR)**	2.5 (0-3)	1 (0-3)	0.42
**Relapse-free interval (wk), Me (IQR)**	11 (4-18)	19 (13-33)	0.60
**FRNS +SDNS, n (%)**	5 (50)	18 (58)	>0.99

Abbreviations: FRNS, frequently relapsing nephrotic syndrome; ITT, intention-to-treat; IQR, interquartile range; Me, median; MMF, mycophenolate mofetil; SDNS, steroid-dependent nephrotic syndrome.

a *P* ≤ 0.05.

Five patients in the MMF group (50%) and 18 patients (58%) in the control group became either steroid-dependent or frequently relapsing (*P* > 0.99). Three patients (9.6%) in the control group and none in the MMF group required treatment for hypertension during induction (*P* = 0.56). Four patients (13%) in the control group and 5 (50%) in the MMF group reported infections, mainly upper respiratory infections, during induction (*P* = 0.01). Two patients (20%) in MMF group reported a bacterial infection (acute otitis media) requiring antibiotics during induction. No serious infections requiring hospitalizations were recorded. Weight gain (Δ body mass index [BMI] and Δ Weight) was not statistically significant in both groups during induction (Table 1). None of the patients reported leukopenia or anemia during induction, blood tests were performed 1 month after initiation of MMF treatment. There were no significant GI side effects in either group based on complaints assessed at every visit.

Per protocol, analysis did not show statistically significant difference in relapse rate during the induction phase and in the median relapse-free interval during 52 weeks of follow-up (Table S1). There were no statistically significant differences in infection rate, hypertension, and weight gain during the induction phase for the patients who completed 52 weeks follow-up (Table S2).

## Discussion

In our small observational study, during the induction phase the relapse rate was higher in the MMF group comparing with the control steroid group; however, by the end of 52 weeks the difference was not statistically significant. By the end of follow-up 70% in the MMF group and 61% in the control group reported at least 1 relapse (Table 2); the rate of relapses in our cohort is consistent with recent published data.^3^^,^^24^ The median relapse-free interval was shorter, whereas the median 52-week relapse rate was higher in the MMF group; the differences did not reach any statistical significance likely because of a small cohort size. To the best of our knowledge, our study is the first to evaluate efficacy of MMF during the induction phase of treatment of new onset steroid-sensitive nephrotic syndrome.

We used a standard 1,200 mg/m^2^ daily dose of MMF and did not measure the pharmacokinetic profiles of mycophenolic acid (12-hour MPA- AUC) in our patients. A plausible explanation is that standard MMF dose may have resulted in subtherapeutic MPA levels in some of the patients.^25^^,^^26^ Significant variability in the activity of inosine monophosphate dehydrogenase has been reported, and higher MPA levels have been associated with better outcomes in autoimmune disorders.^27^^,^^28^ Especially young children with nephrotic syndrome might be underexposed while using standard dosages of MMF.^29^ Monitoring 12-hour MPA-AUC and using higher doses recommended for patients not controlled on standard MMF dose.^3^

Prolonged steroid exposure carries the cumulative risk of side effects.^5^^,^^7^, ^8^, ^9^^,^^11^ The second goal of our study was the minimization of steroid-induced side effects. We did not observe significant differences between the groups in terms of weight gain and BMI. Hypertension rate was higher in patients treated with steroids. No serious infections were reported whereas mild infections were observed more frequently in the MMF group. Both treatment protocols were well tolerated; no dose adjustments were required because of side effects in either group.

All patients in our cohort remained steroid sensitive and by the end of the 52-week follow-up period the relapse-free interval was similar between the groups (Fig 2). All patients with steroid-dependent nephrotic syndrome and frequently relapsing nephrotic syndrome received MMF as a steroid-sparing agent during the follow-up period (Table 2).

Treatment protocols of new onset steroid-sensitive nephrotic syndrome have not changed significantly during the last 50 years.^3^^,^^12^ Neither prolongation nor intensification of initial steroid regimen has changed the subsequent course of steroid-sensitive nephrotic syndrome in terms of risk of frequently relapsing nephrotic syndrome or steroid-dependent nephrotic syndrome and number of relapses.^13^^,^^15^^,^^16^ Shorter induction steroid course was successfully used in steroid sensitive patients with acceptable results.^3^ International Pediatric Nephrology Association guidelines currently recommend using 8-12-week course of steroids for children with new onset nephrotic syndrome.^3^ Our small study confirms that steroids remain the best induction therapy option for pediatric patients with new onset of nephrotic syndrome.

To establish the noninferiority of MMF to steroids, a multicenter trial with substantial sample size is imperative. The primary outcome of such a study should be the relapse rate during 52 weeks follow-up. Based on our results MPA level should be checked 2 weeks after initiation of treatment and MMF dose escalation allowed to achieve higher MPA levels. Secondary outcomes should include steroid and MMF toxicities (weight gain, BMI, hypertension, diarrhea, leukopenia, infectious rate, and quality of life) and total amount and timing of relapses. Using statistical parameters from our study (Table 2), we calculated that 229 patients in each group are required to confirm noninferiority of MMF to steroids (Table S3). Considering the incidence of new nephrotic syndrome of about 2.9 per 100,000 children,^24^ the total amount of children aged 1-12 years (about 47 × 10^6^, based on childstats.gov data), and estimating that 80% are steroid sensitive, 1,360 cases of new steroid-sensitive nephrotic syndrome would be eligible for such study in the United States. Anticipating a 30% participation rate, the study would need the involvement of more than 50% of all children’s hospitals in the United States to recruit the required number of patients within a 2-year timeframe. A prospective interventional study of that scale has never been done previous in the pediatric nephrology field.

A European randomized multicenter trial (INTENT) is underway to compare MMF to steroids as an option during induction phase of treatment for steroid-sensitive nephrotic syndrome.^19^ This study is planning to enroll 340 patients and follow them for 24 months.

Limitations of our study include the low number of patients and prolonged time of enrollment because of single center and reluctance of patients to use a novel therapy (only 24% of screened patients consenting to participate in the MMF group), and lack of MPA pharmacokinetic profiles monitoring. Despite those shortcomings MMF has a potential to be used as an alternative agent in children with new onset nephrotic syndrome especially in those whose high-dose steroids might need to be avoided. Likely, higher doses of MMF are required to achieve similar results to steroids outcomes.

In conclusion, our small cohort of patients treated with MMF reported a higher relapse rate during the induction phase. However, by 12 months of follow-up the relapse rate and relapse-free interval were similar between the groups. All patients tolerated MMF without significant side effects, and those who relapsed remained steroid sensitive. Monitoring MPA-AUC and higher doses of MMF should be considered.

## Acknowledgments

The authors acknowledge Dr Weiss for helping with concept and protocol of the study.
