# Supplementary material for: Mycophenolate Mofetil Versus Prednisone for Induction Therapy in Steroid-Sensitive Idiopathic Nephrotic Syndrome in Children: An Observational Study
Source: Kidney Med. 2023 Dec 10;6(3):100776. doi: 10.1016/j.xkme.2023.100776 (PMC10904994; doi:10.1016/j.xkme.2023.100776)
Supplement: Supplementary File (PDF) — Figure S1; Table S1-S3. [file mmc1.pdf]

Figure S1. Flow diagram

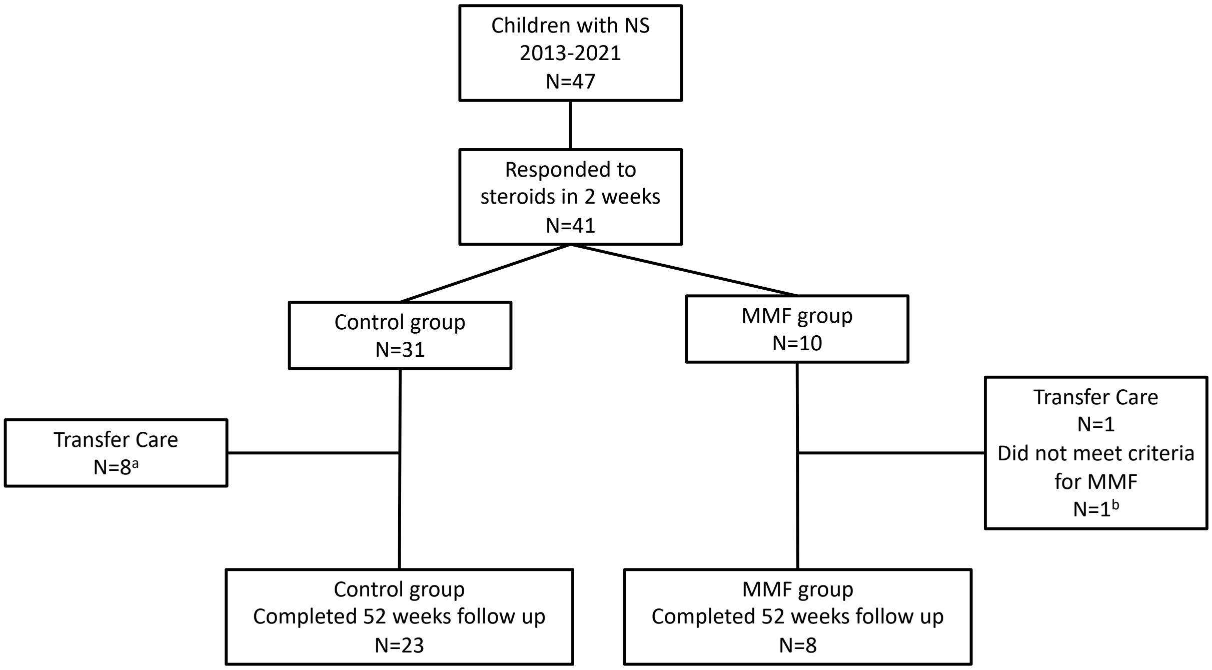

Abbreviations: MMF, mycophenolate mofetil; NS, nephrotic syndrome

a Follow up period was from 2 weeks to 9 months with Me 2 months and IQR (2-6)

b Patient was started on MMF while he had proteinuria with UPC 0.4mg/mg

Table S1. Nephrotic syndrome course in per protocol population

	MMF group (n=8)	Control group (n=23)	P value
**Patients with relapses during induction, n (%)**	2 (25)	0	0.06
**Patients with relapses during 52 w follow up, n (%)**	5 (62)	15 (65)	>0.99
**Relapse Rate, Me (IQR)**	1.5 (0-3)	1 (0-2.5)	0.87
**Relapse Free Interval in weeks, Me (IQR)**	11 (3-25)	21 (13-38)	0.65
**FRNS +SDNS, n (%)**	3 (38)	10 (43)	>0.99

Abbreviations: FRNS, frequently relapsing nephrotic syndrome; IQR, interquartile range; Me, median; MMF, mycophenolate mofetil; SDNS, steroid dependent nephrotic syndrome

*=p<0.05

Table S2. Patients’ characteristics during 10 weeks induction phase (per protocol population)

	MMF group (n=8)	Control group (n=23)	P value
**Age in years, Me (IQR)**	6.7 (3-9)	4.2 (3-6)	0.42
**Male sex, n (%)**	8 (100)	12 (52)	0.03*
**Hypertension, n (%)**	0 (0)	2 (9)	>0.99
**Infections, n (%)**	3 (38)	2 (9)	0.09
**Antibiotics, n (%)**	1 (13)	0	0.26
**∆ BMI, Me (IQR)**	0.1 (-0.3-1.0)	0.3 (-0.1-1.4)	0.73
**∆ Weight in kg, Me (IQR)**	1.5 (0.5-1.9)	1 (0.4-2.5)	0.98

Abbreviations: BMI, body mass index; IQR, interquartile range; Me, median; MMF, mycophenolate mofetil;

*=p<0.05

Table S3. Sample size calculation

**Significance level (alpha)**	5%
**Power (1-beta)**	80%
**Percentage 'success' in control group**	39%
**Percentage 'success' in experimental group**	30%
**Non-inferiority limit, d**	20%
**Sample size required per group**	229
**Total sample size required**	458

Note: Power calculator for binary outcome non-inferiority trial.

https://www.sealedenvelope.com/power/binary-noninferior/
